# Factors influencing COVID-19 testing: a qualitative study in Bhutan

**DOI:** 10.1186/s41256-022-00241-7

**Published:** 2022-04-02

**Authors:** Sonam Yangchen, Solip Ha, Abraham Assan, Tashi Tobgay

**Affiliations:** 1 Institute of Health Partners, Thimphu, Bhutan; 2 Seoul National University Graduate School of Public Health, Seoul, South Korea; 3 Global Policy and Advocacy Network (GLOOPLAN), Accra, Ghana

## Abstract

**Background:**

The COVID-19 pandemic has reaffirmed an all-knowing truth—that health is central in the 2030 Sustainable Development Agenda. To fully control the infection in a community, accurate testing of suspected cases along with effective tracing and appropriate treatment (3Ts) is still crucial to slowing transmission of COVID-19 virus This study explored factors that influenced COVID-19 testing in Bhutan. The lessons learned from this study could serve as a roadmap to strengthen the current respond to COVID-19 and for future outbreaks, particularly in low- and middle-income countries.

**Methods:**

The study employed an exploratory qualitative design. Data collection methods included interviews with key informants with a purposively selected sample of 20 participants. The interview findings were augmented by reviewing both published literature and unpublished documents.For the analysis of qualitative interviews, a hybrid approach of inductive and deductive coding and theme development was conducted to analyze findings. A tailored version of the WHO Health System Framework incorporating the Essential Public Health Function was used to guide data interpretation.

**Results:**

Political will through the influence and leadership of the King of Bhutan played a crucial role in raising attention to the problem, and provision of adequate financial and technical relief to ensure that all people irrespective of their socioeconomic status do not pay to get tested of COVID-19. A compassionate leadership, Whole of Society approach is backed by the scientific community, functional health systems and community-based approaches, use of information technology for awareness creation and improved surveillance system, and fast-tracked COVID-19 testing service delivery.

**Conclusion:**

Bhutan’s success relied greatly on multi-sectoral and systematic approach during policy design, implementation and monitoring, and active collaborative efforts involving consultation and engagements with a broad range of local (community members), national and global actors for accelerated COVID-19 testing. These efforts were made possible through improved health governance and leadership at all levels of the society.

## Introduction

The COVID-19 pandemic is an unparalleled global disaster. There have been more than 220 million confirmed cases of COVID-19, including 4.57 million deaths, reported to World Health Organization (WHO) as of 8 September 2021 [1]. Despite the importance of vaccination, a return to the “*first principles*” approach of accurate test, effective tracing, and appropriate treatment (3Ts) is still crucially important to slowing transmission [2]. Extensive testing efforts have been key to some countries’ responses with some of the lowest fatality rates in the world [3]. Increasing testing capacity restricts COVID-19 to specific areas, providing targeted interventions and contain further spread of infection The global supply of testing tools and reagents is a finite resource and inherently limited. Without judicious and well-reasoned testing strategies, demand will far outstrip supply [4]. While geopolitically powerful countries use their considerable economic and political resources to procure supplies for testing instruments and reagents for themselves, prices surge due to increasing demand for the test kits [5]. This leaves smaller and poorer nations reliant on donors for supplies [6]. Countries have rolled out vaccination to prevent hospitalization and severe disease to halt transmission of the virus. However, there are challenges with regard to vaccine production, affordability, allocation and deployment [7]. Some low and middle income countries (LMICs) have been slow in rolling out the vaccination mainly due to supply crunch and insufficient funds [8]. Concerns regarding the safety and efficacy of COVID-19 vaccines, as well as myths and misinformation, on social media has added to vaccine hesitancy [8].

Bhutan is a LMIC located in South East Asia with an estimated 682,000 people dispersed across 38,394 square km [9]. It is a mountainous and landlocked country and has a democratic constitutional monarchy system of governance, which was started in 2008. As per the World Bank, the country’s Gross Domestic Product per capita was USD 3122 in 2020 [10]. Bhutan’s health care system is based on the principles of Primary health care (PHC) with 186 Primary Health Centers, 542 outreach clinics and 52 sub-posts manned by Health Assistants (HA) at the grass-root level, 49 general hospitals at major towns and district level. Two regional referral hospitals (one in eastern and the other one in central Bhutan), and a National Referral Hospital provide tertiary care services. However, the country does not have any pharmaceutical or biotechnology manufacturing facilities. All medicines and medical supplies are procured from outside the country.

The first case of COVID-19 in Bhutan was reported on 5 March 2020, and the number had increased to 2596 by 8 September 2021 [11] with only two deaths. Despite being a resource-constrained country, as of 31 July 2021, Bhutan had successfully vaccinated over 90% adults. Bhutan has one of the highest testing rates in the South East Asia region [12]. This study examines the barriers and facilitators to implementing COVID-19 testing in Bhutan by investigating the country’s early response. Despite the existence of known barriers and facilitators to testing, these are described in the literature only in commentary or opinion pieces globally; primary research studies that explore the effect of such barriers and facilitators in context are lacking. This current study adds new knowledge about barriers and facilitators of COVID-19 testing by analyzing the perspectives of communities, implementers, and policymakers in a South East Asian LMIC. It aims to fill a gap in the literature by deepening understandings about barriers and facilitators to COVID-19 testing. The lessons learnt from Bhutan can be beneficial to small and resource poor countries to contain current pandemics and well as strategizing future prevention of pandemics.

## Methods

### Study design

Qualitative research methods were undertaken to explore the barriers and facilitators related to COVID-19 testing implementation in Bhutan. Semi-structured interviews were conducted with 20 purposively sampled key informants from January to March 2021. Informed consent was sought from all key informants and where consent was given, interviews were recorded. Interviews were conducted virtually and lasted between 30 and 60 min. To triangulate interview data and understand the context to conceptualize key themes from collected data, a desk review was conducted for documents consisting of policy documents, national emergency preparedness plan, national COVID-19 situation reports, and published literature on Bhutan’s COVID-19 response.

### Participants and sampling

Interviews were conducted with 20 key informants comprising of policymakers, implementers, frontline workers, and community members. Key informants from policy maker and implementer group were selected considering their position, and influence in decision making, implementation, and evaluation of COVID-19 testing in Bhutan. Key informants from frontline worker group were selected to represent different health facility types, and community members were selected to understand the community perspective. The informants included members of the parliament, civil society organizations, National Technical Advisory Group Members, National Task Force Members and community representatives. This wide range of key informants from policymakers, field staff, and community members maximize the diversity of information and their perspectives. The sample size was kept open to reach data saturation defined as no more new information within the different key informant groups and researchers were confident that data gathered were sufficient, consistent, and of quality to meet the objectives. All researchers agreed on four general principles and concepts regarding data saturation; no new data, no new themes, no new coding, and the ability to replicate the study.

### Data collection tool and procedures

The research team invited all eligible persons to participate in the study via email with the consent form, which outlined the purpose of the study, including their roles and responsibilities as research participants. All interviews were audio-recorded after obtaining verbal consent and conducted virtually through a Zoom platform, from January until March 2021 by authors SY, SH and AA. Notes were also taken where necessary. Flexible semi-structured interview guides were designed for different categories of interviewees. Guides were amended depending on participant’s position and involvement in COVID-19 testing and covered elements drawn from the WHO Health System Framework and the Essential Public Health Functions (EPHF) [13, 14].The content of the EPHF frameworks is divided into two categories: cross-cutting (horizontal) functions, based roughly on the building blocks approach to health systems; and service-based (vertical) functions comprising the traditional public health services provided by modern health systems [13, 14]. These two frameworks formed the basis of our thematic analysis. Interview questions covered: general perceptions of challenges and facilitators of COVID-19 testing in Bhutan, the number of testing and collected samples per day, supply chain process, human/material/financial resources, surveillance systems, and prioritizing process for vulnerable populations.

### Data processing and analysis

Data were transcribed verbatim. Most interviews were conducted in English. Interview with few participants, who do not speak English, were conducted in the local Bhutanese language (*Dzongkha)* by author SY and transcribed into English. These translations were verified by professional translators to enhance accuracy.

An inductive coding and theme development was conducted to analyze findings. Transcripts were analyzed by the research team using a coding scheme developed from our thematic framework. We employed the framework by following these steps: 1) familiarization 2) generating initial codes 3) indexing 4) generating themes 5) defining and naming themes. Throughout analysis, we remained open to accommodate emerging themes [15].

Coding was conducted manually, and a codebook was developed to examine and code the data. We conducted an open breakdown of themes to identify all existing themes in the study (open coding) with 130 codes. Next, we extracted, compared, categorized, and created interconnections, and discussed relevant codes which were associated with the study objectives. 16 subthemes emerged. Finally, core categorical themes were selected, analyzed and compared with existing literature to ensure the reliability, validity and comprehensiveness of our findings.

## Results

In Table 1, key informant characteristics are summarized. Interviews were conducted with 20 key informants comprising policymakers, implementers, frontline workers, and community members.

**Table 1:** Characteristics of key informants interviewed

Key informants	Characteristics of key informants	Total
Policymakers (PM)	3 central MoH Officials 1 Member of Parliament 1 COVID-19 Technical Advisory Group (TAG) members	5
Implementers (IM)	2 Testing managers from facility 2 Public health program managers 2 Lab officers	6
Frontline workers (FW)	2 Frontline worker from Regional Referral Hospitals 1 Frontline worker from Community Health Center 2 Frontline worker from District health centers	5
Community members (CM)	2 Community volunteers 1 Villager 1 Community leader	4
Total		20

From the interview data analysis, five overarching themes related to facilitators of COVID-19 testing, and three themes for barriers emerged. Themes for facilitators are governance and leadership; resources, health service delivery, the Whole of Society and digitalization of health services. Themes for barriers are geographical barriers, lack of resources, and misconceptions about COVID-19 (Table 2).

**Table 2:** Categories, sub-categories and themes

	Categories	Sub-categories	Themes
Facilitators	1. Governance and leadership	1.1 Political leadership	Compassionate leadership and guidance Personal assistance in provision of relief measures Improved community participation led by the King Political will and support to get the mass tested Health as a high-priority sector in national governance
		1.2 Community leadership	Designated volunteer and champions in the community High community willingness to be tested Community engagement to provide key information and services Adherence to COVID-19 guidelines Engagement of religious heads and leaders in advocacy programs
		1.3 Health governance system	Adherence to WHO recommendations National COVID-19 response plan/protocol Effective early preparedness and planning Evidence-based testing protocols and strategies Effective response to procurement challenges through health governance Lessons learned from the previous pandemic, MERS
	2. Resources	2.1 Capacities of in-service personnel	Positive attitude of health professionals Training for community members, medical students to fill the human resource gap Improved interpersonal relationships across agencies Improved confidence of health professionals to work in high-risk areas with Personal Protective Equipment (PPEs)
		2.2 Financial and material Resources	Government's financial support to get tested of COVID-19 Effective resource mobilization Door-to-door sampling strategy eased testing procedures Sufficiency in supplies—PPEs, test kits, cold chain, Rapid expansion of testing sites and facilities
	3. Health service delivery	3.1 Primary Health Care (PHC)	Stronger PHC health system targeting Universal Health Coverage (UHC) Establishment of flu clinics to screen suspected COVID cases away from the hospitals 24 hours health helpline for all, including ambulatory services
		3.2 Surveillance	System in place to trace and treat vulnerable population Online reporting and monitoring system Ability to identify defaulters and get them tested Effective monitoring system to ensure the adequacy of materials for testing Zoning system to facilitate easy access for testing at the place of dwelling
	4. Whole of Society Approaches (WOS)		Active engagement of intergovernmental organizations and private sectors, armed forces, civil society organizations and volunteer groups
	5. Use of digital technology		Good data management system that updates daily COVID-19 situation Systematic and comprehensive collection of samples using the national demographic data Geographic information system (GIS) mapping to identify a high-risk population
Barriers	1. Geographical barriers	1.1 Porous border with neighboring countries	Potential risks and outbreaks from neighboring countries
		1.2 Poor transport networks	Delays in the transportation of supplies to remote districts Poor transport networks due to season changes
	2. Lack of human resources		Lack of epidemiologists and biomedical engineers Shortage of health professionals and mid-level managers for conducting COVID-19 testing Challenges of using online technology when training health professionals in all regions of Bhutan
	3. Misconceptions about COVID-19 testing	3.1 Misconceptions about symptoms of COVID-19	Misconceptions about symptoms of COVID-19 as symptoms of seasonal flu
		3.2 Fear of nasal swab for COVID-19 testing	Misconceptions about negative health implications associated with nasal swab due to misinformation through mass media

### Facilitators to COVID-19 testing

#### 1. Governance and leadership

##### 1.1. Political leadership

Ninety five percent (n = 19) of the informants pointed to the leadership of the King of Bhutan to support for the lives and livelihood of the people. Based on our findings, there were strong perceptions of the King’s compassion as a significant factor in unifying the people to come together in the fight against COVID-19, which contributed to enhancing manpower and resources for testing for COVID-19. The leadership uplifted the motivation of the health workers and community with the objective to combat the pandemic. Informants reported that King's guidance strongly influenced a sense of collective responsibility:

"Everybody is tired now, some people have been working day and night but the commitment is still going strong. There is no dearth of dedication because of the encouragement from the King. His Majesty always makes sure that he comes around, meets and encourages people". – MoH policymaker 1.

Several community members and implementers highlighted that government's response to COVID-19 was a transparent and evidence-based whole-of-society approach which allowed long-term strategic cooperation. Bhutan’s leaders guided the formulation of a National Preparedness and Response Plan well before the first case was detected with the objective to enhance the health sector’s capacity to enhance surveillance, detect, control and prevent COVID-19 outbreak in the country. The National Preparedness and Response Plan included specific roles for all government ministries, private sector, local authorities, civil society organizations and NGOs.

Informants highlighted that the one facilitator of Bhutan's successful response to COVID-19 was that a number of senior-level officials, including the Prime Minister, Minister for Foreign Affairs, and Minister for Health were all health professionals with public health backgrounds:

"Strong leadership is from our monarch and our current government, because if you look into the cabinet, you will find that three of them are medical doctors and public health experts so that was an advantage for us.”- a National-level policymaker 1.

##### 1.2. Community leadership

Eighty five percent (n = 17) of the informants confirmed that people in the community made various contributions to the increased engagement in nationwide activities to respond to COVID-19. Most informants stated that community members contributed to providing agricultural products to the government during nationwide lockdown for distribution to frontline workers and marginalized populations. Local leaders were involved in monitoring the home-quarantined individuals, providing essential supplies, enforcing public health protocols in the community, administrating lockdowns, and coordinating arrangements, encouraging people to get tested:

"We have local leaders known as Gup and they provide basic health services in our community. Also, even though our MoH provides awareness program through social media groups, local leaders help us to disseminate information related to availing testing". - Community health center frontline worker 1.

Eighty percent (n = 8) of implementers and community members mentioned that religious practice was a great help to relieve COVID-19-related stress and tensions, including facilitating testing procedures. High compliance to COVID-19 protocols was maintained in Bhutan in part because people rely on religious leaders for advice. In Bhutan, Buddhism is the state religion and is a core spiritual anchor for Bhutanese. Religious leaders have contributed to response to COVID-19 testing, and religious institutions were also important hubs for advocacy programs of COVID-19 prevention. Most of informants emphasized that many religious leaders and monastic institutions led many prayers and ceremonies for the prevention of the outbreak. In addition, they were actively engaged in advocating COVID-19 prevention programs:

“We believe in Buddhism and many monastic institutions lead prayers or ceremonies for the prevention of outbreak and for the welfare of the country. They help us by cooperating, and letting people follow all the protocols including coming for testing”- District health center frontline worker 2.

##### 1.3. Health governance system

Seventy seven percent (n = 7) of community members and policymakers stated that in response to COVID-19, the MoH developed and implemented strategies to ensure continuity of essential services during the pandemic. They emphasized that no individuals were put under financial hardship to access health services in Bhutan as all health services including testing were provided by the State and people did not have to pay to avail the health services. In addition to free testing and medical services, all meals and accommodation were provided freely by the state at the designated quarantine and isolation facilities. The government reprioritized and consolidated savings from non-essential activities from all sectors to invest these into the COVID- 19 response.

Based on previous regional experiences with MERS,the Bhutan Influenza Pandemic Preparedness Plan (BPPRP) was initiated based on an established strategy to deal with infectious disease outbreaks and forms part of the ‘Health Sector Disaster Contingency Plan.’ This focuses on broad and robust surveillance and evidence-based risk assessment that can capture disease outbreaks from emerging pathogens in general. MoH and District Health offices collectively worked for planning and responding to pandemics at the national and local level:

“One of the success factors to response to COVID-19 is that we had learned from previous experiences during the era of MERS coronavirus, respiratory syndrome outbreak in the Middle East in the early 2000s. So when this COVID-19 came in, we could prepare guidelines and standards to circulate the information to all implementers.”-MoH policymaker 3.

#### 2. Resources

Bhutan rapidly expanded testing centers across the country in all 20 districts. Considering testing as the main strategy to prevent and contain epidemics, RT-PCR testing facilities were expanded from one at the Royal Center for Disease Control (RCDC) to 5 centers across the country during the pandemic period. This proved effective for handling sample surge during community outbreaks and mass testing [12].

The MoH initiated training programs for existing health professionals in areas of epidemiology, health system and procurement surveillance, and response and emergency preparedness. To address dire shortage of human resources, task shifting, and task sharing proved to be vital during lockdown, testing, vaccination rollout and all other activities to optimally utilize the technical staff [16].

##### 2.1. Capacities of in-service personnel

The findings from interviews confirmed that training for community members, medical and nursing students from Universities and health professionals were helpful to fill the human resource gap. Bhutan has limited numbers of doctors, nurses, and laboratory technologists, and no infectious disease specialists, virologists, or immunologists. The training contributed to a positive impact on improving the confidence of health professionals and interpersonal relationships across the agencies to work together even in high-risk areas:

“Due to shortage of human resources, we trained health professionals, frontline workers and University students so that they can be ready for the sample collection for COVID-19 testing. We also sent few expert doctors and lab officials from the capital to train all the lab and other health workers in other districts”. - Central laboratory implementer 1.

##### 2.2. Financial and material resources

Eighty five percent (n = 17) of informants emphasized the importance of providing governmental financial support for people to get tested for COVID-19, and effective resource mobilization for the COVID-19 response. Frontline workers and implementers pointed out that the door-to-door sampling strategy eased testing procedures. They confirmed that resources were allocated by the government and it played a leading role in ensuring sufficient supplies and rapid expansion of testing sites and facilities. The government and the King granted financial assistance to vulnerable population who were affected by the pandemic and lost their jobs:

“One important thing I want to emphasize is that the government and the king has granted financial assistance to anybody who has been affected by the pandemic, who has lost their jobs due to the pandemic". - Member of Parliament 1.

#### 3. Health service delivery

##### 3.1. Primary health care (PHC)

Informants confirmed that a total of 55 flu clinics were set up separately in the country away from main hospital building for separating patients with suspected or confirmed COVID-19 and patients presenting with respiratory illnesses. The main goal was to prevent compromising hospitals with COVID-19 cases and ensure continuity of other healthcare services. Health services, including testing services were delivered even to remote villages using existing system of PHC networks. A 24 hours health helpline, including ambulatory services was provided for all patients across the country:

"During the lockdowns, the service was delivered at home. We didn't really have to go look for services. There were mobile clinics and we had health workers going around and ensuring that everybody gets the services. So personally, I did not face any barrier.” –Community member 1.

“We have set up a dedicated hotline for the elderly and patients. We also ensure that they get the regular health service on time. We try to have health workers reach medicines to them. We have hotlines for the general population and for them.”- MoH policymaker 1.

“The strengths of Bhutanese health system is a functional primary health care along with WHO's principles of universal health coverage and health system strengthening to guide policy directions.”- MoH policy maker 2.

##### 3.2. Surveillance

In addition to the existing National Early Warning Alert and Response Surveillance system, the Royal Centre for Disease Control established a COVID-19–integrated influenza surveillance system. Most frontline workers stated that the government has systems to trace and treat vulnerable populations and online reporting and monitoring systems enabled them to identify defaulters and get them tested. Informants also pointed out that this effective monitoring system helped ensure the adequacy of materials for testing such as test kits and PPEs.

The implementation of a zoning system during the lockdown period facilitated active surveillance. The zoning system helped geographically define zones according to risk of COVID-19 transmission. Those in low-risk areas such as rural and high-land areas were allowed to return to normal life early on, whereas high-risk areas such as Thimphu, the capital city of Bhutan and the southern districts bordering India with a high number of transmissions had longer periods of lockdown. A policymaker from MoH noted that,

“We have active surveillance system, and if there is a positive case, we do contact tracing and we get all the contacts and a mix of facility quarantine and home quarantine. This is regularly updated by frontline workers and reflected to the online monitoring system.”

#### 4. Whole of society approaches

Bhutan followed a Whole-of-the-Society approach to combat pandemic with a slogan, “our *Gyenkhu”* (our responsibility). The Whole of Society Approaches acknowledges the contribution and important role played by all relevant stakeholders, including individuals, communities, intergovernmental organizations and religious institutions, civil society, academia, the media, voluntary associations and, where appropriate, the private sector and industry [17].

Ninety five percent (n = 19) of the informants said that active engagement of intergovernmental organizations, private sectors, armed forces, civil society and national volunteer groups were a key to a successful response to COVID-19 during the lockdown. Especially, government offices, hospitals, Institutions like the Bhutan Red Cross Society, medical university and the national referral hospital, and civil society organizations supported the MoH’s efforts and advocated health advice on testing, physical distancing, use of face masks, and avoidance of crowds. Private and business sectors also donated testing kits and supplies. The armed forces took a lead role in enforcing COVID-19 protocols.

A frontline worker from a regional referral hospital pointed out that,

“In terms of whole of the society, other ministries and NGOs are supporting MoH in the fight against the COVID-19 and even during this outbreak by promoting Covid-19 response programs I think our health system is well prepared to handle this outbreak and the pandemic with all different sectors.”

#### 5. Use of digital technolog﻿y

Technology was widely used for risk communication, reaching out to patients for teleconsultations and even for pandemic response operations. Some implementers and policymakers stated that the one successful facilitator of COVID-19 prevention was good data management system. The MoH shared all data with frontline workers and implementers and the number of tests, and their results are reflected in the centralized system that made it possible for up-to-date monitoring of health needs of the population. As a result, most of the implementers could monitor progress of testing and update the results to the integrated COVID influenza surveillance system.

The Geographic Information System (GIS) mapping was used to identify high-risk populations. The MoH developed and implemented a tracking app called, *Druk-trace* that allowed quick scanning of a response code to register one's presence at a location. This was useful for contact tracing and people without smart phones were registered in a logbook maintained at all public locations including offices, shops, and public transport:

“All data are centralized so whatever the test and report comes, they are put into the same system so they are a centralized data base and all the reports are shared in the system and we could get access, and generate status of testing and then we could report to the ministry and various other agencies.”- Central laboratory implementer 2.

### Barriers to COVID-19 testing

#### 1. Geographical barriers

##### 1.1. The porous border with neighboring countries

Ninety five percent (n = 19) of the informants expressed their concerns about porous borders, which could be potential sources of the outbreak. The country partly shares borders with India and China. Due to the free movement of people and travelers across the border with India, the entry and exit gates are exposed to the risk for COVID-19 transmission and have challenges in conducting strict surveillance after the outbreak of COVID-19.

Bhutan’s porous land borders were a cause for concern among policymakers and health professionals, who feared that unchecked migration and transport between countries could spread the virus quickly:

“These porous borders could be possible sources of the infection coming to this country. We classified southern border with India as high-risk areas. The South was declared high-risk areas. If you want to travel from the designated high-risk districts, or if you want to travel to northern districts from South, you must undergo a mandatory one-week quarantine and then test to come out.”—MoH policy maker 3.

##### 1.2. Poor transport network

According to eighty percent (n = 16) of informants, Bhutan’s transportation network poses a separate set of challenges. Due to poor transportation infrastructure, shipment of supplies such as test kits and PPEs for health workers in remote districts was delayed. Frontline workers and policymakers pointed out the importance of improved transportation infrastructure to respond to COVID-19:

''There were transportation barriers, especially in the southern districts when there is heavy rainfall. This makes it difficult to access the communities during testing. Again, it is about a five to six-hour journey from the testing center in Thimphu.” -Testing facility implementer 1.

A policymaker from MoH also quoted that,

“Initially we had challenges. Especially when RT-PCR tests were done, we had established a lab in the national referral hospital, so initially we had to transport our sample taken to the national lab, in the capital, it's about a minimum five-hour drive. This was time-consuming, and the results were also delayed.”

#### 2. Lack of human resources

Lack of trained specialists, especially epidemiologists, biomedical engineers, mid-level managers for conducting COVID-19 testing were highlighted by informants as one of the leading barriers of COVID-19 testing. Also, challenges of using online technology when training health professionals and workers in all regions in Bhutan were commonly mentioned by most frontline workers and implementers.

A frontline worker from the national regional referral hospital said that,

“We are in shortage of the expertise like epidemiologists. We have to sometimes ask experts in other countries virtually, and we have some epidemiologists who have come from animal health sector, so in future, we must develop that. We also have inadequate mid-level managers and biomedical engineers to manage this pandemic situation”.

#### 3. Misconceptions about COVID-19 testing

##### 3.1. Misconceptions about symptoms of COVID-19

Informants pointed out some misconceptions among people about COVID-19 symptoms. Some people do not want to get tested because they consider them as seasonal flu symptoms. In addition, some of them still believe that COVID-19 is just like the flu.

A policymaker from MoH said that,

“There are some people who have certain symptoms of seasonal flu, but they don't want to come forward to get tested. Most people who have flu-like symptoms think that they may have just flu, not COVID-19. During flu season, it is very challenging to encourage people to get tested. Also, few people believe that COVID-19 is no worse than seasonal flu.”

##### 3.2. Fear of nasal swab for COVID-19 testing

Some of the community members who experienced the nasopharyngeal swab for COVID-19 testing commented that “*the testing is not the most pleasant thing in the world, and I felt somebody was touching the throat.*” This testing experience is openly shared with more people through diverse mass media, and for this reason, some people fear the nasal swab and they are reluctant to get tested:

“Due to fear of testing, people will not come for testing. For example, when you go out to be vaccinated, you might see kids waiting for their turns scared of the needle. Like this, some people are afraid of getting nasal swabs.” - MoH policymaker 3.

## Discussion

This qualitative study on COVID-19 testing from Bhutan, a small, land-locked, resource-constrained country, explored facilitators and barriers to scale-up Covid-19 testing using semi-structured interviews and a purposive sampling approach. We summarized our findings into two main themes, facilitators and barriers with sub themes; governance and leadership, resources, health service delivery, Whole of Society, use of digital technology, geographical settings,and misconceptions about Covid-19 testing.

The findings of this study will help us to scale up Covid-19 testing, especially in resource poor settings. Besides, a compassionate leadership, Whole of ASociety Approach backed by the scientific community, functional health systems and community based approaches have been identified successful factors for active response to the pandemic. This will help more countries develop context specific strategy to respond to Covid-19 in the future. Further, this study will provide policy implications and insights into how governments, communities and other sectors can collaboratively contribute to strengthening health policies and systems.

The leadership of King in Bhutan was a key motivational driver and moral yardstick that made people from all walks of life exhibit a collective responsibility to promote testing as the core strategy to combat COVID-19. Bhutan has always followed a decentralized policy with communities at the central of any decisions making process and the five years plans are developed with a participatory approach [18]. Bhutan’s governance principles are guided by the principles of Gross National Happiness (GNH). The GNH measures community participation in the government’s decision and community vitality as a measure of community happiness and governments performance in creating a conducive environment for happiness [18]. This may have facilitated community participation for enhanced community testing strategy. This was further facilitated by involving religious leaders who reinforced and reassured the need to follow health advice during such pandemics. In addition, cross-government task forces were established at the Sub-district level, District level, Regional level, and National Level, each having clear roles and responsibilities. In terms of whole of government, the task force even included the military and Ministry of Foreign Affairs with the disaster management department as the secretariate to the team. This ensured harmonization and coordination of the actions, including testing strategies as prevention, containment, and opening of lockdown. This clearly showed that leadership of political leaders as well as religious leaders and cross sectoral collaborations between different sectors is essential to effectively respond to the pandemic.

In Bhutan, health is a basic human right, guaranteed and protected by the constitution. Appropriate measures like free testing and pick up and drop of patients requiring emergency medical care during the lockdowns were put in place. These measures ensured that the people are not denied health services including testing for COVID-19 due to financial hardship. Studies have shown that financial support from government can have positive impact to encourage more people to get tested and increase their willingness towards Covid-19 testing [19–21]. There are also studies that identified logistical barriers to testing such as geographic, socioeconomic, and structural disparities [20, 21], while others report a lack of knowledge about how or where to access a test [20–22].Furthermore, attitudes towards testing play a large role in test avoidance; distrust, fear and stigma associated with COVID-19 and associated public health responses pose a significant challenge; this is equally applicable to diverse contextual settings [21, 22].Despite successful implementation of COVID-19 testing strategy, Bhutan faced some challenges such as geographical barriers, lack of human resources and medical supplies and misconceptions on COVID-19. Bhutan’s landlocked geographical position stood as a barrier during the procurement of medical drugs and equipment, especially when all countries were faced with shortages. Even though Bhutan has a central procurement management system to monitor medical supplies, it is difficult to transport to other districts due to poor infrastructure and transportation networks. Bhutan is constrained of human workforce working in the health sector. The HR situational report conducted by WHO and MoH revealed that Bhutan’s availability of doctors, nurses and midwives is 22.5 per 10,000 populations, below the WHO SDG threshold, established at 44.5 per 10,000 population [23]. Lack of adequate human resources, especially epidemiologists, biomedical engineers, were leading barriers to implementing COVID-19 testing in Bhutan. Logistical constraints exist when distributing tests to populations in rural areas with poor road networks, which compromised timely transport of the sample to laboratories.

Preliminary policy implications were drawn from our findings and observations. One strategy to increase testing capacity and reduce the procurement burden is to strengthen and support local manufacturing of supplies with a collaborative approach such as whole of government and society. For example, South Korea has established public–private partnerships to develop diagnostic test kits and expedited approval of these tests by the Korean Ministry of Food and Drug Safety (MFDS) enabling rapid scale-up of testing [22]. Further, a collaboration between governments, manufacturers in LMIC, and international organizations has been shown to be key to address shortage of medical supplies [24]. With the collective actions, inedaquate human resources as well as materials issues were well addressed in Bhutan.

Infodemic has been one of the biggest challenge in combating COVID-19 globally [25]. Mis-and disinformation through social media was in abundance even in Bhutan. To overcome these challenges, first, public health communicators may benefit from using story-telling and other practical tools that confront misinformation. Second, concerted efforts need to strengthen laws to address the use of data and personal health information. Finally, initiatives such as WHO’s evidence-informed policy network and intervention by the United Nations, can bring together policymakers, researchers and civil society that can be replicated at national or district levels [26].

Given the rapidly evolving nature and evidence on COVID-19, massive testing for Covid-19 is still considered very important, as widespread community transmission has become entrenched in many countries. Thus, technical knowledge on Covid-19 should be shared especially with LMICs accompanied by know-how transfer of fast, efficient and accurate testing strategy to ensure the quality of test results. Also, building the regional cooperation will improve investment incentives to facilitate testing centers, research institutions and hospitals and will develop good manufacturing practice (GMP) related skills. This also will ensure pandemic preparedness for future pandemics in the long term [27].

Our study has certain strengths and limitations. This is one of the first attempts to identify barriers and facilitators of the COVID-19 testing in Bhutan using a qualitative method. The identified barriers and facilitators can help health authorities to analyze gaps to implement testing strategies efficiently. We conducted semi-structured interviews across four different groups of informants, which led to richer exchanges and provided opportunities to probe important emerging narratives. The paper illuminates the perspectives and experiences of Bhutan’s Government Technical advisory Group for COVID-19 and health workers across all levels of health systems, which are not always considered together in publications.

On the other hand, our study holds a few limitations. First, the sample size of key informants was small, and the virtually conducted interviews and COVID-19 restrictions (lockdown) limited our ability to conduct field observation and Focus Group Discussions (FGDs) as planned. Second, the rapidly evolving nature of the pandemic and the changing national context is different from the time of data collection to that of the manuscript development phase. Considering the focus on the testing, the richness of information and opportunity for deeper exploration on these topics may have been lost.

## Conclusion

Having a leadership with high moral authority and compassion was seen as the single most driver for a successful testing strategy in Bhutan. This was further augmented through a combination of multifaceted factors such as: the Whole of Society Approach to health; active community participation; Bhutan's pragmatic tactic in delivering on the promise of universal health coverage through primary health care strategy; the use of information technology for awareness creation and improving surveillance system; all fast-tracked COVID-19 testing service delivery.

## Data Availability

The data used and analysed during the current study cannot be shared publicly for the privacy of individuals that participated in the study.The data are available from the corresponding author on reasonable request. All papers included in this study are available in the ‘References’ section.

## Abbreviations

BPPRP: Bhutan Influenza Pandemic Preparedness Plan
EPHF: Essential Public Health Functions
FGDs: Focus Group Discussions
GIS: Geographic Information System
HA: Health Assistant
LMICs: Low and middle income countries
MFDS: Ministry of Food and Drug Safety
MoH: Ministry of Health
PHC: Primary health care
PPE: Personal Protective Equipment
RCDC: Royal Center for Disease Control
WoS: Whole of Society
